# Epilepsy duration is an independent factor for electrocardiographic changes in pediatric epilepsy

**DOI:** 10.1002/epi4.12519

**Published:** 2021-07-19

**Authors:** See Wai Chan, Leslie A. Dervan, Robert Scott Watson, Anne E. Anderson, Yi‐Chen Lai

**Affiliations:** ^1^ Department of Pediatrics Baylor College of Medicine Houston TX USA; ^2^ Department of Pediatrics University of Washington Seattle WA USA; ^3^ Center for Clinical and Translational Research Seattle Children’s Research Institute Seattle WA USA; ^4^ Center for Child Health, Behavior, and Development Seattle Children’s Research Institute Seattle WA USA

**Keywords:** cardiac, ECG, epilepsy, pediatric, temporal

## Abstract

**Objective:**

Cardiac alterations represent a potential epilepsy‐associated comorbidity. Whether cardiac changes occur as a function of epilepsy duration is not well understood. We sought to evaluate whether cardiac alterations represented a time‐dependent phenomenon in pediatric epilepsy.

**Methods:**

We retrospectively followed pediatric epilepsy patients without preexisting cardiac conditions or ion channelopathies who had history of pediatric intensive care unit admission for convulsive seizures or status epilepticus between 4/2014 and 7/2017. All available 12‐lead electrocardiograms (ECGs) from these patients between 1/2006 and 5/2019 were included. We examined ECG studies for changes in rhythm; PR, QRS, or corrected QT intervals; QRS axis or morphology; ST segment; or T wave. Data were analyzed using multivariable models containing covariates associated with ECG changes or epilepsy duration from the univariate analyses.

**Results:**

127 children with 323 ECGs were included in the analyses. The median epilepsy duration was 3.9 years (IQR 1.3‐8.4 years) at the time of an ECG study and a median of 2 ECGs (IQR 1‐3) per subject. The clinical encounters associated with ECGs ranged from well‐child visits to status epilepticus. We observed changes in 171 ECGs (53%), with 83 children (65%) had at least 1 ECG with alterations. In a multivariable logistic regression model adjusting for potentially confounding variables and accounting for clustering by patient, epilepsy duration was independently associated with altered ECGs for each year of epilepsy (OR: 1.1, 95% CI: 1.0‐1.2, *P* = .002). Extrapolating from this model, children with epilepsy durations of 10 and 15 years had 2.9 and 4.9 times the odds of having ECG changes, respectively.

**Significance:**

Cardiac alterations may become more common with increasing epilepsy duration in select pediatric epilepsy patients. Future studies are needed to determine the potential clinical implications and the generalizability of these observations.

**Key points**

- ECG alterations are common in a select cohort of pediatric epilepsy patients.
- Epilepsy duration is an independent risk factor for ECG alterations.
- Children with 10 and 15 years of epilepsy have 3 and 5 times the odds of having ECG changes compared to those with <1 year of epilepsy.
- Studies are needed to determine the clinical implications and the generalizability in the pediatric epilepsy population.

## 1 INTRODUCTION

Childhood‐onset epilepsy is a common neurological condition affecting more than 20 million children worldwide, with a disproportionately high rate of years living with disability.^1^ In addition to the developmental and cognitive impairments associated with epilepsy, cardiac alterations may constitute a potential epilepsy‐associated comorbidity, including interictal and periictal rhythm disturbances,^2^, ^3^, ^4^, ^5^, ^6^, ^7^ corrected QT interval (QTc) changes,^8^, ^9^, ^10^, ^11^, ^12^, ^13^ and ventricular conduction and repolarization abnormalities.^14^, ^15^, ^16^, ^17^, ^18^, ^19^ These cardiac alterations can be detrimental, supported by the increased arrhythmogenic potential under conditions of periictal hypoxemia^13^, ^20^ and seizure‐associated fatal and near‐fatal arrhythmias.^21^, ^22^, ^23^ Despite these associations of cardiac abnormalities with potentially deleterious consequences, whether cardiac changes occur as a function of epilepsy duration is not well understood.

In small cohorts of adult epilepsy patients and in children with Dravet syndrome, worsening indices of cardiac electrophysiology with increasing epilepsy duration suggest that cardiac electrical alterations may be progressive in epilepsy.^19^, ^24^ Using standard 12‐lead electrocardiogram (ECG), we previously reported that children with epilepsy admitted to the pediatric intensive care unit (PICU) for convulsive status epilepticus had increased odds for ECG alterations.^25^ In this study, we seek to investigate whether ECG abnormalities are associated with duration of time since epilepsy diagnosis in a cohort of pediatric epilepsy subjects with history of PICU admission for convulsive seizures and status epilepticus.

## 2 METHODS

### 2.1 Patient selection and data collection

The Baylor College of Medicine Institutional Review Board approved this retrospective, longitudinal study with a waiver of consent. The study cohort was selected from children with epilepsy who had a history of admission to the Texas Children's Hospital PICU between 4/2014 and 7/2017 with primary diagnosis of convulsive seizures or status epilepticus. Children were included if they had at least one standard 12‐lead ECG study following their diagnosis of epilepsy in the electronic medical record between 1/2006 and 5/2019 and excluded if they had preexisting cardiac conditions or known ion channelopathies.

Patient demographics and the age of epilepsy onset were collected. For each ECG study, we extracted chronic seizure types, maintenance antiseizure medications (ASM), inotropic medications, reason for the clinical encounter, indication for ECG, location where the ECG study was performed, whether there was a documented seizure episode within 24 hours prior to the ECG study, and laboratory data. When applicable, we also estimated the time interval between the most recent clinical or electrographic seizure and the ECG study. The age at epilepsy diagnosis was determined based on either neurology service documentation or the first appearance of International Classification of Diseases, 9th revision (ICD‐9) diagnostic or 10th revision (ICD‐10) codes for epilepsy. Refractory epilepsy was determined based on the documentation by the neurology service, operationally defined as failure of adequate trials of two appropriately chosen and tolerated ASMs.^26^ All ECGs were read by a pediatric cardiac electrophysiologist per our institutional clinical standards. We extracted these official ECG interpretations to identify heart rate, measurements of PR, QRS, and QTc intervals, rhythm abnormalities (atrial or ventricular arrhythmias, heart block, or junctional rhythms), PR interval shortening or prolongation, QRS axis abnormalities (right or left axis deviation, northwest axis, indeterminate axis), QRS morphologic or interval abnormalities (right or left bundle branch block, incomplete right or left bundle branch block, interventricular conduction delay, or low voltage), QTc interval shortening or prolongation, ST segment abnormalities (nonspecific changes, early repolarization, ischemic changes), or T‐wave abnormalities (inverted T wave, nonspecific changes, notched T wave, late peaking T wave). An ECG study was considered to be abnormal if abnormalities in rhythm, PR interval, QRS axis, QRS interval, QTc interval, ST segment, or T wave were present.

### 2.2 Statistical analyses

The primary outcome was the odds of having an abnormal ECG as function of epilepsy duration. We reported patient demographics, clinical and laboratory data, and details of ECG abnormalities using descriptive statistics. In univariate analyses, we evaluated associations between clinical characteristics and presence of abnormal ECG using the Pearson chi‐square test for categorical variables and the Mann‐Whitney *U* test and generalized linear modeling for continuous variables. We evaluated univariate associations between clinical characteristics and epilepsy duration using linear regression with robust standard errors. Multivariable logistic regression, adjusting for potentially confounding variables and accounting for multiple observations (clustering) by patient, was used to evaluate the association of epilepsy duration with the odds of having an ECG abnormality. Prespecified model covariates included reason for the clinical encounter and number of ASMs based on clinical concerns for confounding. Additional covariates associated with epilepsy duration and with abnormal ECG with *P* < .1 in univariate analyses were also included in the multivariable model. The final model therefore also included the chronic seizure type, presence of abnormal electrolytes or acidosis, and ionotropic therapy. Age at ECG was excluded, as it was highly collinear with epilepsy duration, with older patients having longer durations of epilepsy. Since age at epilepsy onset was not associated with the presence of abnormal ECG, the association between abnormal ECG and age was attributable to increasing epilepsy duration. We performed a sensitivity analysis restricted to the first ECG study following epilepsy diagnosis and constructed a multivariable model using the same methodological approach. Statistical computations were performed using STATA, version 14.2 (StataCorp LP). Two‐sided *P* values of <.05 were considered statistically significant.

## 3 RESULTS

Between 4/2014 and 7/2017, 427 children were admitted to the PICU with the primary diagnosis of convulsive seizures or status epilepticus. Two hundred forty‐four of the 427 children had epilepsy (57%). Of those, 102 children had preexisting cardiac condition/known channelopathy (n = 38) or no ECG study (n = 64) and were excluded. One hundred forty‐two had the diagnosis of epilepsy and had at least one standard 12‐lead ECG study between 1/2006 and 5/2019. Fifteen children with epilepsy had ECG studies only prior to the diagnosis and were excluded. The remaining 127 children had ECG studies (n = 323) at or following the diagnosis of epilepsy and were included in the study.

Males predominated (57%), and the median age of epilepsy diagnosis was 1.8 years (IQR 0.5‐5 years) with a median of 2 ECGs (IQR 1‐3) per subject following the diagnosis of epilepsy (Table 1). Sixty‐five children had refractory epilepsy. The median epilepsy duration at the time of a given ECG study was 3.9 years (IQR 1.3‐8.4 years) (Table 1). Reasons for the clinical encounters, ECG indications and locations, chronic seizure types, and number and names of maintenance ASMs are summarized in Table 1. Increased seizure frequency/status epilepticus was the most common reason for the clinical encounter associated with an ECG study (34%), followed by routine office visits (27%). The PICU (35%) and outpatient setting (27%) represented the most common locations where ECG studies were performed. The two most common seizure types were focal onset‐impaired awareness/focal onset to bilateral tonic‐clonic seizures (33%), followed by generalized onset tonic‐clonic seizures (16%). The most common maintenance ASM was levetiracetam (51%), followed by carbamazepine/oxcarbazepine (23%), zonisamide (23%), and clobazam (19%) (Table 1).

**TABLE 1:** Demographic and clinical characteristics of 127 patients with epilepsy

	N (%) or median [IQR]
Male gender	73 (57)
Race	
White	32 (25)
Black	23 (18)
Hispanic	67 (53)
Asian	5 (4)
Age at epilepsy diagnosis (years)	1.8 [0.5‐5]
Number of ECGs per subject	2 [1‐3]
Epilepsy duration at the time of ECG^a^	3.9 [1.3‐8.4]
Refractory epilepsy	65 (51)
Reason for clinical encounters^a^	
Increased seizure frequency/status epilepticus	110 (34)
Office visits	86 (27)
Respiratory concerns	40 (12)
New‐onset seizure	19 (6)
Other	68 (21)
ECG locations^a^	
Outpatient	88 (27)
Emergency center	84 (26)
Regular inpatient floor	34 (11)
Intensive care unit	114 (35)
Unknown	3 (1)
ECG indications^a^	
Tachycardia/Bradycardia	60 (19)
Arrhythmias	59 (18)
Heart evaluation	40 (12)
Evaluate QT/ST segment	18 (6)
Seizures	36 (11)
CNS abnormalities	24 (7)
Medication evaluation	16 (5)
Respiratory concerns	4 (1)
Other	66 (20)
Chronic seizure types^a^, ^b^	
Focal onset	
Aware	6 (2)
Impaired awareness	90 (28)
Focal to bilateral tonic‐clonic	13 (4)
Generalized onset	
Tonic‐clonic	53 (16)
Tonic, clonic, or atonic	87 (27)
Myoclonic and others	43 (13)
Epileptic spasm	26 (8)
Absence	9 (3)
None of the above/not available	96 (30)
Maintenance ASM^a^, ^b^	
Levetiracetam	166 (51)
Carbamazepine/Oxcarbazepine	74 (23)
Zonisamide	73 (23)
Clobazam	60 (19)
VNS/Surgery	46 (14)
Clonazepam	45 (14)
Topiramate	38 (12)
Phenobarbital	33 (10)
Rufinamide	26 (8)
Lacosamide	24 (7)
Lamotrigine	22 (7)
Valproic acid	20 (6)
Vigabatrin	11 (3)
Ketogenic diet	9 (3)
Other	48 (15)
Not available/missing	31 (10)
Number of maintenance ASMs^a^	
0	20 (6)
1	81 (25)
2	82 (25)
3	43 (13)
4+	65 (20)
Not available/missing	31 (10)

Abbreviations: ASM, antiseizure medication; CNS, central nervous system; ECG, electrocardiogram; IQR, interquartile range; VNS, vagal nerve stimulator.

^a^ Among the 323 individual ECGs obtained among the 127 patients following the diagnosis of epilepsy.

^b^ Some patients exhibited multiple seizure types and were on multiple ASMs. Percentages do not add to 100% accordingly.

**FIGURE 1:**
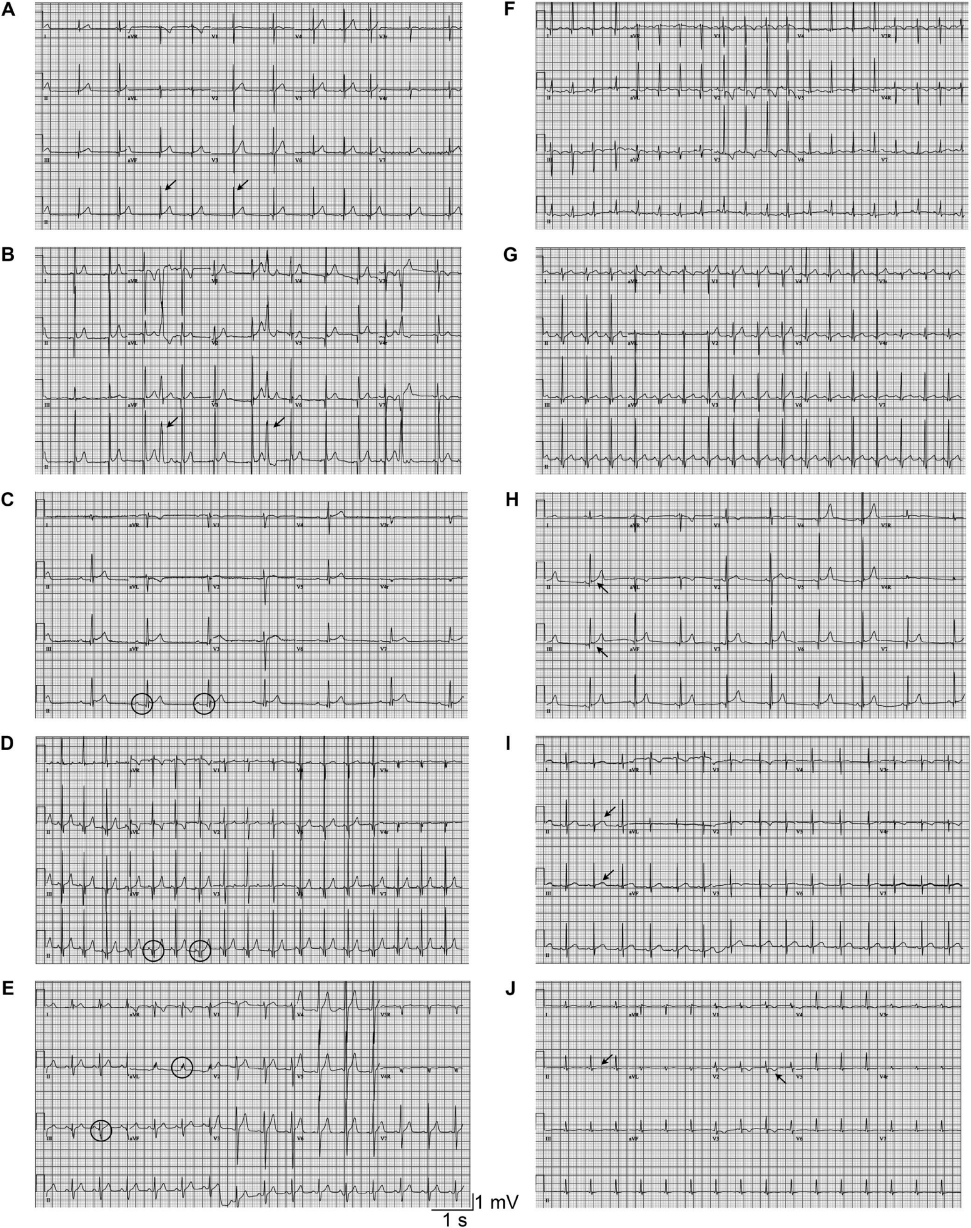
ECG abnormalities in pediatric epilepsy. Sample ECG tracings that illustrate diverse abnormalities. A, Junctional rhythm (arrows). B, Premature ventricular contractions (arrows). C, 1st degree AV block (circles). D, Short PR interval (circles). E, Interventricular conduction delay (circles). F, Left axis deviation. G, Right axis deviation. H, Nonspecific ST segment changes (arrows). I, Nonspecific T‐wave abnormalities (arrows). J, Flatten T wave and inverted precordial T wave (arrows)

Eighty‐three of 127 children (65%) had at least 1 abnormal ECG study, and 171 of 323 ECG studies (53%) exhibited 1 or more abnormalities (Table 2; Figure 1). We observed a spectrum of ECG abnormalities including rhythm disturbances and alterations in interval duration, axis, and morphology. Changes in the ST segment and T‐wave abnormalities were the most common abnormalities observed (Table 2).

**TABLE 2:** ECG characteristics of 323 ECGs obtained in 127 patients with epilepsy

	N (%)
ECG abnormality present	
By patient (n = 127)	83 (65)
By individual ECG study (n = 323)	171 (53)
Rhythm abnormalities	
Premature ventricular contractions	4 (1)
Junctional rhythm	1 (0.3)
Abnormal PR interval	
Long PR interval (1st degree AV block)	7 (2)
Short PR interval	8 (2)
QRS axis	
Right axis deviation	14 (4)
Left axis deviation	9 (3)
QRS morphology	
Interventricular conduction delay	5 (2)
Right bundle branch block	4 (1)
Low voltage	4 (1)
Incomplete right bundle branch block	2 (0.6)
Widening	1 (0.3)
Prolonged QTc interval	7 (2)
ST segment abnormalities	
Nonspecific changes	60 (19)
Early repolarization	9 (3)
T‐wave abnormalities	
Nonspecific changes	98 (30)
Inverted T wave	5 (2)
Inverted T wave +nonspecific changes	3 (1)

Abbreviations: ECG, electrocardiogram; QTc, corrected QT interval.

Univariate analyses revealed that epilepsy duration was associated with increased likelihood of having an abnormal ECG study (unadjusted RR for each year of epilepsy: 1.05, 95% CI: 1.03‐1.07, *P* < .0005) (Table 3). Abnormal ECG studies were also associated with age at which ECG was obtained, chronic seizure type, reason for clinical encounter, the presence of abnormal electrolytes or acidosis, and the presence of inotropes. Age of epilepsy onset, diagnosis of refractory epilepsy, presence of recent seizures, interval between seizures and ECG, location where ECG studies were obtained, and number of chronic ASMs were not associated with abnormal ECG studies (Table 3). Epilepsy duration was associated with seizure semiology, increasing number of chronic ASMs, refractory epilepsy, age at which ECG was performed, and electrolyte abnormalities or acidosis (Table S1).

**TABLE 3:** Univariate analysis of clinical factors associated with abnormal ECG

	Abnormal ECG (n = 171)^a^	Normal ECG (n = 152)^a^	RR	95% CI	*P*
Epilepsy duration (years)^b^	6 [2‐10]	3 [1‐6]	1.05	1.03‐1.07	<.0005
Age at ECG (years)^b^	9 [4‐15]	6 [2‐11]	1.03	1.02‐1.05	<.0005
Age of epilepsy onset (years)^b^	1.3 [0.5‐5]	1 [0.5‐4]	1.00	1.00‐1.00	.6
Seizure semiology					
Focal impaired awareness or focal to bilateral tonic‐clonic	61 (36)	38 (25)			.01
Generalized tonic‐clonic	24 (14)	20 (13)			
Generalized tonic	33 (19)	20 (13)			
Other/missing	53 (31)	74 (49)			
Number of maintenance ASMs					
0	10 (6)	10 (8)			.9
1	46 (28)	35 (27)			
2	46 (28)	36 (28)			
3	22 (14)	21 (16)			
4+	39 (24)	26 (20)			
Refractory epilepsy	82 (48)	68 (45)	1.06	0.9‐1.3	.6
Reason for clinical encounter					
Routine visit	42 (25)	44 (29)			.03
New seizure	7 (4)	12 (8)			
Increase in seizure/SE	62 (36)	48 (32)			
Respiratory	29 (17)	11 (7)			
Other	31 (18)	37 (24)			
ECG locations					
Outpatient	39 (23)	49 (32)			.1
Emergency Center	42 (25)	42 (28)			
Regular inpatient floor	19 (11)	15 (10)			
Pediatric intensive care unit	68 (40)	46 (30)			
Unknown	3 (2)	0 (0)			
History of seizures in prior 24 h^c^					
No	76 (51)	63 (53)			.7
Yes	73 (49)	55 (47)			
Seizures to ECG interval (min)^d^	262 [102‐489]	255 [85‐393]			.8
Abnormal electrolytes or acidosis^e^	59 (35)	28 (18)	1.4	1.2‐1.7	.001
Inotropes	1 (1)	9 (5)	1.7	1.4‐2.2	.017

Note

Categorical variables were analyzed using Pearson chi‐square test. Seizures to ECG interval was analyzed using a Mann‐Whitney *U* test.

Abbreviations: ASM, antiseizure medication; CI, confidence interval; ECG, electrocardiogram; RR, relative risk; SE, status epilepticus.

^a^ Results presented as n (%) or median [interquartile range].

^b^ RR and CI estimated using generalized linear models; the RR describes the additional average risk of abnormal ECG associated with each additional year of age or epilepsy duration.

^c^ Normal ECG: n = 118 and abnormal ECG: n = 149.

^d^ Normal ECG: n = 40 and abnormal ECG: n = 58.

^e^ Includes any of the following: serum potassium <3 or >6 mmol/L, magnesium <2 mg/dL, phosphorus <2.5 mg/dL, total calcium <7.5 mg/dL, or base excess <−5.

In a multivariable logistic regression model, epilepsy duration was independently associated with the presence of ECG abnormalities (OR: 1.1, 95% CI: 1.0‐1.2, *P* = .002) adjusting for abnormal electrolytes or acidosis, ionotropic therapy, number of ASMs, reason for clinical encounter, chronic seizure type, and accounting for multiple observations (clustering) by patient (Table 4). Extrapolating from this model, children with 5 years of epilepsy duration have an estimated 1.7 times the odds of having abnormal ECGs compared to children within 1 year of epilepsy diagnosis. Children with 10 years and 15 years of epilepsy duration have an estimated 2.9 times and 4.9 times the odds of having abnormal ECGs, respectively.

**TABLE 4:** Multivariable logistic regression analysis evaluating the association of epilepsy duration with odds of abnormal ECG^a^

	OR	95% CI	*P*
Epilepsy duration (years)	1.1	1.0‐1.2	.002
Abnormal electrolytes or acidosis	1.8	0.9‐3.4	.09
Inotropes	4	0.3‐46	.3
Number of maintenance ASMs	0.9	0.7‐1.1	.2
Reasons for clinical encounter			
New seizure	0.7	0.2‐2.6	.6
Increasing seizure/SE	1.3	0.6‐2.7	.5
Respiratory	2.6	0.9‐7.6	.1
Other	0.8	0.4‐1.8	.6
Seizure semiology			
Focal impaired awareness or focal to bilateral tonic‐clonic	1.4	0.7‐2.9	.3
Generalized tonic‐clonic	1.2	0.5‐2.8	.7
Generalized tonic	1.8	0.9‐3.8	.1

Abbreviations: ASM, antiseizure medication; CI, confidence interval; ECG, electrocardiogram; OR, odds ratio; SE, status epilepticus.

^a^ Accounting for multiple observations (clustering) by patient.

In a sensitivity analysis restricted to the first ECG study following epilepsy diagnosis, epilepsy duration and electrolyte abnormalities or acidosis were associated with abnormal ECG studies. In a multivariable model adjusting for abnormal electrolytes or acidosis, number of maintenance ASMs, and reason for clinical encounter, longer epilepsy duration had higher odds of abnormal ECG (OR = 1.1, 95% CI: 1.0‐1.3, *P* = .06; Table S2), although this finding did not reach statistical significance.

## 4 DISCUSSION

In this study, we retrospectively assessed a select group of children with epilepsy and history of ICU admission who underwent formal ECG assessment for different indications, in different clinical locations, at different times during their epilepsy. We found that epilepsy duration is an independent risk factor for ECG changes in these 127 children with a history of PICU admission for convulsive seizures or status epilepticus suggesting that cardiac alterations may be a time‐dependent phenomenon in pediatric epilepsy patients with a history of severe seizure exacerbation that requires ICU level care.

Epilepsy‐associated cardiac electrical alterations represent a well‐known systemic comorbidity in adult epilepsy^4^, ^5^, ^6^, ^7^, ^8^, ^9^, ^10^, ^11^, ^12^, ^13^, ^14^, ^15^, ^20^, ^27^, ^28^ and to a lesser extent in childhood‐onset epilepsy.^2^, ^3^, ^16^, ^25^ Additionally, recent observations in small cohorts of adult epilepsy individuals and children with Dravet syndrome suggest that epilepsy‐associated cardiac electrical alterations may worsen over time. Specifically, adults with long‐standing epilepsy have been shown to exhibit interictal and postictal pathological T‐wave alternans, an indicator of cardiac electrical instability and a risk marker for sudden cardiac death^18^, ^19^; whereas adults with newly diagnosed epilepsy do not exhibit pathological T‐wave alternans.^19^ Similarly, epilepsy duration of 5 years or greater is associated with longer QTc intervals and worse spatial‐temporal ventricular repolarization heterogeneity in children with Dravet syndrome.^24^ Our findings in this cohort of pediatric epilepsy patients without ion channelopathy or cardiac conditions add to the growing clinical evidence for cardiac electrical alterations as a function of epilepsy duration.

We found that ECG changes were common in this cohort of pediatric epilepsy patients. Nonspecific ST segment and T‐wave changes represented the most common alterations with the prevalence of 19% and 30%, respectively. In comparison, the prevalence of nonspecific ST elevation and T‐wave changes is approximately 10% and 5% in over 48,000 school‐age children participating in an ECG screening program.^29^ In adult population‐based studies, the prevalence of early repolarization (characterized by the ST segment alterations) and nonspecific ST‐T changes is approximately 6% and 10%, respectively.^30^, ^31^ Although nonspecific ECG changes are generally considered to have indeterminate clinical significance, they may nevertheless reflect underlying cardiac electrophysiological changes such as increased ventricular repolarization complexity,^32^ which could have future clinical impact. Accordingly, population‐based, longitudinal studies in adults have revealed nonspecific ST segment and T‐wave changes to be independent risk factors for cardiac mortality decades following the ECG studies.^30^, ^31^ Together, these observations suggest that minor ST/T‐wave abnormalities may represent early indicators of altered cardiac electrophysiology and risk biomarkers. The clinical implication of the observed ECG changes and the underlying cardiac electrophysiological alterations in pediatric epilepsy are currently unknown. Future prospective studies are needed to further characterize cardiac electrophysiological alterations represented by the minor ECG changes and to evaluate their potential long‐term impact in the overall pediatric epilepsy population.

The observed association between epilepsy duration and ECG changes may be attributable to several factors. Univariate analyses revealed that chronic seizure type, abnormal electrolytes or acidosis, and the presence of inotropes were associated with ECG alterations. Other potential contributing factors include the number of ASM and their mechanisms of action, as many ASMs modulate ion channel activities and could thereby impact cardiac electrophysiology.^33^, ^34^ Adjusting for these potential contributing factors in our analysis, epilepsy duration remained independently associated with ECG changes. The observed ECG alterations could also simply represent an acute effect of seizures or status epilepticus. However, in this cohort, these changes were not associated with the presence of a seizure episode or time since last recorded seizure, and the prevalence of ECG studies with alterations was similar across various clinical locations where ECGs were obtained (outpatient, emergency center, inpatient, PICU). Together, these findings suggest that, while well‐described ECG alterations do arise from seizures or status epilepticus, the observed ECG changes in this study are not likely due to transient seizure‐related phenomena.

Because some ECG features evolve with age in the pediatric population,^35^ these ECG alterations may simply represent the expected developmental changes. We utilized the official reports by the pediatric cardiac electrophysiologists who reviewed all ECG studies as part of routine clinical practice at our institution. Their evaluation would have accounted for the expected age‐dependent ECG norms. Furthermore, we did not observe an association between age of epilepsy onset and abnormal ECG studies. Together, these findings suggest that the observed ECG alterations do not simply reflect age‐dependent changes.

Several mechanisms may underlie epilepsy duration and ECG abnormalities. For instance, aberrant sympathovagal balance with sympathetic predominance, represented by alterations in heart rate variability, has been extensively described in epilepsy.^27^ Persistent sympathetic predominance in adults with long‐standing epilepsy can adversely affect cardiac function such as increased cardiac stiffness and decreased exercise tolerance.^36^ Similarly, myocardial molecular remodeling suggestive of persistent sympathetic predominance has been described in the animals with early as well as long‐standing epilepsy.^37^, ^38^ These observations indicate that epilepsy may adversely affect the heart through aberrant autonomic regulation leading to myocardial remodeling. Future studies are needed to gain further mechanistic insights into the association of epilepsy duration with cardiac abnormalities.

This cohort of pediatric epilepsy patients with a history of PICU admission and ECG studies performed for specific clinical indications represents a selective group, which limits the generalizability of our findings. Studies in more general pediatric epilepsy cohorts will be necessary to further ascertain whether epilepsy duration is an independent factor associated with cardiac alterations in the overall pediatric epilepsy population. Although we excluded children with known ion channelopathy, we did not explore other genetic influences that might also contribute to the observed ECG changes. Furthermore, we could not exclude cumulative seizure burden as a factor that underlies the association between epilepsy duration and abnormal ECG studies. Similarly, we could not evaluate the combined effects of chronic seizure type and epilepsy duration on the observed ECG changes or the cumulative ASM exposure as a factor that might underlie the association between epilepsy duration and abnormal ECG studies. The sensitivity analysis failed to meet statistical significance. However, the magnitude and direction of the odds ratio were similar to the final, larger‐sample multivariable model. Therefore, these results are consistent and support our primary findings.

This study provides the first description of epilepsy duration as an independent risk factor for ECG changes in a highly selective group of pediatric epilepsy patients without cardiac conditions or ion channelopathies. Our findings add to the emerging clinical evidence suggesting that cardiac alterations may be a time‐dependent phenomenon. Future prospective studies are needed to determine the incidence of ECG alterations as a function of epilepsy duration in the overall pediatric epilepsy population, provide mechanistic insights into progressive cardiac alterations, and determine their clinical implications.

## CONFLICT OF INTEREST

The authors do not have any conflicts of interest to disclose.

## ETHICAL APPROVAL

We confirm that we have read the Journal's position on issues involved in ethical publication and affirm that this report is consistent with those guidelines.

## Supporting information

Tab S1‐S2

## References

[1] Olusanya BO, Wright SM, Nair MKC, Boo NY, Halpern R, Kuper H, et al. Global burden of childhood epilepsy, intellectual disability, and sensory impairments. Pediatrics. 2020;146:e20192623. 32554521 10.1542/peds.2019-2623 PMC7613313

[2] Harnod T, Yang CC, Hsin YL, Shieh KR, Wang PJ, Kuo TBJ. Heart rate variability in children with refractory generalized epilepsy. Seizure. 2008;17:297–301. 17977751 10.1016/j.seizure.2007.09.002

[3] Mayer H, Benninger F, Urak L, Plattner B, Geldner J, Feucht M. EKG abnormalities in children and adolescents with symptomatic temporal lobe epilepsy. Neurology. 2004;63:324–8. 15277628 10.1212/01.wnl.0000129830.72973.56

[4] Nei M, Sperling MR, Mintzer S, Ho RT. Long‐term cardiac rhythm and repolarization abnormalities in refractory focal and generalized epilepsy. Epilepsia. 2012;53:e137–40. 22709423 10.1111/j.1528-1167.2012.03561.x

[5] Romigi A, Albanese M, Placidi F, Izzi F, Mercuri NB, Marchi A, et al. Heart rate variability in untreated newly diagnosed temporal lobe epilepsy: evidence for ictal sympathetic dysregulation. Epilepsia. 2016;57:418–26. 26813146 10.1111/epi.13309

[6] Sivakumar SS, Namath AG, Tuxhorn IE, Lewis SJ, Galán RF. Decreased heart rate and enhanced sinus arrhythmia during interictal sleep demonstrate autonomic imbalance in generalized epilepsy. J Neurophysiol. 2016;115:1988–99. 26888110 10.1152/jn.01120.2015 PMC4869507

[7] Toth V, Hejjel L, Fogarasi A, Gyimesi C, Orsi G, Szucs A, et al. Periictal heart rate variability analysis suggests long‐term postictal autonomic disturbance in epilepsy. Eur J Neurol. 2010;17:780–7. 20100226 10.1111/j.1468-1331.2009.02939.x

[8] Brotherstone R, Blackhall B, McLellan A. Lengthening of corrected QT during epileptic seizures. Epilepsia. 2010;51:221–32. 19732135 10.1111/j.1528-1167.2009.02281.x

[9] Drake ME, Reider CR, Kay A. Electrocardiography in epilepsy patients without cardiac symptoms. Seizure. 1993;2:63–5. 8162376 10.1016/s1059-1311(05)80104-9

[10] Neufeld G, Lazar JM, Chari G, Kamran H, Akajagbor E, Salciccioli L, et al. Cardiac repolarization indices in epilepsy patients. Cardiology. 2009;114:255–60. 19672064 10.1159/000233236

[11] Surges R, Scott CA, Walker MC. Enhanced QT shortening and persistent tachycardia after generalized seizures. Neurology. 2010;74:421–6. 20124208 10.1212/WNL.0b013e3181ccc706 PMC2872619

[12] Surges R, Adjei P, Kallis C, Erhuero J, Scott CA, Bell GS, et al. Pathologic cardiac repolarization in pharmacoresistant epilepsy and its potential role in sudden unexpected death in epilepsy: a case‐control study. Epilepsia. 2010;51:233–42. 19817816 10.1111/j.1528-1167.2009.02330.x

[13] Seyal M, Pascual F, Lee CY, Li CS, Bateman LM. Seizure‐related cardiac repolarization abnormalities are associated with ictal hypoxemia. Epilepsia. 2011;52:2105–11. 21906052 10.1111/j.1528-1167.2011.03262.x PMC3203996

[14] Nei M, Ho RT, Abou‐Khalil BW, Drislane FW, Liporace J, Romeo A, et al. EEG and ECG in sudden unexplained death in epilepsy. Epilepsia. 2004;45:338–45. 15030496 10.1111/j.0013-9580.2004.05503.x

[15] Nei M, Ho RT, Sperling MR. EKG abnormalities during partial seizures in refractory epilepsy. Epilepsia. 2000;41:542–8. 10802759 10.1111/j.1528-1157.2000.tb00207.x

[16] Akalin F, Tirtir A, Yilmaz Y. Increased QT dispersion in epileptic children. Acta Paediatr. 2003;92:916–20. 12948066 10.1080/08035250310003550

[17] Schomer AC, Nearing BD, Schachter SC, Verrier RL. Vagus nerve stimulation reduces cardiac electrical instability assessed by quantitative T‐wave alternans analysis in patients with drug‐resistant focal epilepsy. Epilepsia. 2014;55:1996–2002. 25470430 10.1111/epi.12855

[18] Strzelczyk A, Adjei P, Scott CA, Bauer S, Rosenow F, Walker MC, et al. Postictal increase in T‐wave alternans after generalized tonic‐clonic seizures. Epilepsia. 2011;52:2112–7. 21933179 10.1111/j.1528-1167.2011.03266.x

[19] Pang TD, Nearing BD, Krishnamurthy KB, Olin B, Schachter SC, Verrier RL. Cardiac electrical instability in newly diagnosed/chronic epilepsy tracked by Holter and ECG patch. Neurology. 2019;93:450–8. 31477610 10.1212/WNL.0000000000008077

[20] Park KJ, Sharma G, Kennedy JD, Seyal M. Potentially high‐risk cardiac arrhythmias with focal to bilateral tonic‐clonic seizures and generalized tonic‐clonic seizures are associated with the duration of periictal hypoxemia. Epilepsia. 2017;58:2164–71. 29105057 10.1111/epi.13934

[21] Espinosa PS, Lee JW, Tedrow UB, Bromfield EB, Dworetzky BA. Sudden unexpected near death in epilepsy: malignant arrhythmia from a partial seizure. Neurology. 2009;72:1702–3. 19433745 10.1212/WNL.0b013e3181a55f90

[22] Ferlisi M, Tomei R, Carletti M, Moretto G, Zanoni T. Seizure induced ventricular fibrillation: a case of near‐SUDEP. Seizure. 2013;22:249–51. 23312350 10.1016/j.seizure.2012.12.008

[23] Lanz M, Oehl B, Brandt A, Schulze‐Bonhage A. Seizure induced cardiac asystole in epilepsy patients undergoing long term video‐EEG monitoring. Seizure. 2011;20:167–72. 21183363 10.1016/j.seizure.2010.11.017

[24] Lyu SY, Nam SO, Lee YJ, Kim G, Kim YA, Kong J, et al. Longitudinal change of cardiac electrical and autonomic function and potential risk factors in children with dravet syndrome. Epilepsy Res. 2019;152:11–7. 30870727 10.1016/j.eplepsyres.2019.02.018

[25] Ali W, Bubolz BA, Nguyen L, Castro D, Coss‐Bu J, Quach MM, et al. Epilepsy is associated with ventricular alterations following convulsive status epilepticus in children. Epilepsia Open. 2017;2:432–40. 29430560 10.1002/epi4.12074 PMC5800777

[26] Kwan P, Arzimanoglou A, Berg AT, Brodie MJ, Allen Hauser W, Mathern G, et al. Definition of drug resistant epilepsy: consensus proposal by the ad hoc Task Force of the ILAE Commission on Therapeutic Strategies. Epilepsia. 2010;51:1069–77. 19889013 10.1111/j.1528-1167.2009.02397.x

[27] Lotufo PA, Valiengo L, Benseñor IM, Brunoni AR. A systematic review and meta‐analysis of heart rate variability in epilepsy and antiepileptic drugs. Epilepsia. 2012;53:272–82. 22221253 10.1111/j.1528-1167.2011.03361.x

[28] Poh MZ, Loddenkemper T, Reinsberger C, Swenson NC, Goyal S, Madsen JR, et al. Autonomic changes with seizures correlate with postictal EEG suppression. Neurology. 2012;78:1868–76. 22539579 10.1212/WNL.0b013e318258f7f1 PMC3369522

[29] Yoshinaga M, Iwamoto M, Horigome H, Sumitomo N, Ushinohama H, Izumida N, et al. Standard values and characteristics of electrocardiographic findings in children and adolescents. Circ J. 2018;82:831–9. 29199265 10.1253/circj.CJ-17-0735

[30] Daviglus ML, Liao Y, Greenland P, Dyer AR, Liu K, Xie X, et al. Association of nonspecific minor ST‐T abnormalities with cardiovascular mortality: the Chicago Western Electric Study. JAMA. 1999;281:530–6. 10022109 10.1001/jama.281.6.530

[31] Tikkanen JT, Anttonen O, Junttila MJ, Aro AL, Kerola T, Rissanen HA, et al. Long‐term outcome associated with early repolarization on electrocardiography. N Engl J Med. 2009;361:2529–37. 19917913 10.1056/NEJMoa0907589

[32] Lux RL, Hilbel T, Brockmeier K. Electrocardiographic measures of repolarization revisited: why? what? how? J Electrocardiol. 2001;34(Suppl):259–64. 11781965 10.1054/jelc.2001.28909

[33] Aurlien D, Gjerstad L, Taubøll E. The role of antiepileptic drugs in sudden unexpected death in epilepsy. Seizure. 2016;43:56–60. 27886630 10.1016/j.seizure.2016.11.005

[34] Kramer DB, Mihatov N, Buch KA, Zafar SF, Ruskin JN. Case 4–2020: a 52‐year‐old woman with seizure disorder and wide‐complex tachycardia. N Engl J Med. 2020;382:457–67. 31995694 10.1056/NEJMcpc1913471

[35] Uygur O, Aydogdu A. Normal electrocardiogram values of healthy children. Turk Pediatri Ars. 2019;54:93–104. 31384144 10.14744/TurkPediatriArs.2019.04568 PMC6666356

[36] Fialho GL, Wolf P, Walz R, Lin K. Increased cardiac stiffness is associated with autonomic dysfunction in patients with temporal lobe epilepsy. Epilepsia. 2018;59:e85–90. 29697139 10.1111/epi.14084

[37] Brewster AL, Marzec K, Hairston A, Ho M, Anderson AE, Lai YC. Early cardiac electrographic and molecular remodeling in a model of status epilepticus and acquired epilepsy. Epilepsia. 2016;57:1907–15. 27555091 10.1111/epi.13516 PMC5545890

[38] Lai YC, Li N, Lawrence W, Wang S, Levine A, Burchhardt DM, et al. Myocardial remodeling and susceptibility to ventricular tachycardia in a model of chronic epilepsy. Epilepsia Open. 2018;3:213–23. 29881800 10.1002/epi4.12107 PMC5983128

