# Supplementary material for: Epilepsy duration is an independent factor for electrocardiographic changes in pediatric epilepsy
Source: Epilepsia Open. 2021 Jul 19;6(3):588–96. doi: 10.1002/epi4.12519 (PMC8408606; doi:10.1002/epi4.12519)
Supplement: Supplementary file 1 — Tab S1‐S2 [file EPI4-6-588-s001.docx]

**Supplemental Table 1. Univariate analysis of clinical factors associated with epilepsy duration.**

|  | **Coefficient^a^** | **95% CI** | ***p*** |
| --- | --- | --- | --- |
| Age at ECG (years) | 0.6 | 0.5-0.7 | <0.0005 |
| Seizure semiology  Focal impaired awareness or focal to bilateral tonic clonic  Generalized tonic-clonic  Generalized tonic  Other/missing | ref.  0.2  -0.1  -3.2 | -  -1.5-1.9  -1.8-1.7  -4.3- -2.0 | -  0.8  0.9  <0.0005 |
| Number of maintenance ASMs  0  1  2  3  4+ | ref.  1.0  3.1  4.4  3.6 | -  -0.8-2.8  1.2-5.0  2.0-6.8  1.5-5.6 | -  0.3  0.001  <0.0005  0.001 |
| Refractory epilepsy | 2.6 | 1.6-3.6 | <0.0005 |
| Reasons for clinical encounter  Routine visit  New seizure  Increase in seizure/SE  Respiratory  Other | ref.  -1.7  -0.3  0.1  0.3 | -  -3.9-0.4  -1.6-0.9  -1.9-2.1  -1.2-1.7 | -  0.1  0.6  0.9  0.7 |
| Abnormal electrolytes or acidosis^b^ | 1.6 | 0.4-2.8 | 0.009 |
| Inotropes | 2.4 | -1.2-6.0 | 0.2 |

^a^ Regression coefficient from linear regression with robust standard errors, representing the estimated average additional years of epilepsy associated with presence of that factor.

^b^ Includes any of the following: serum potassium < 3 or > 6 mmol/L, magnesium < 2 mg/dL, phosphorus < 2.5 mg/dL, total calcium < 7.5 mg/dL, or base excess < -5.

ASM: anti-seizure medication. CI: confidence interval. ECG: electrocardiogram. SE: status epilepticus.

**Supplemental Table 2. Multivariable logistic regression analysis evaluating the association of epilepsy duration with odds of abnormal ECG restricted to the first ECG study following epilepsy diagnosis^a^**

|  | **OR** | **95% CI** | ***p*** |
| --- | --- | --- | --- |
| Epilepsy duration (years) | 1.1 | 1.0-1.3 | 0.06 |
| Abnormal electrolytes or acidosis | 2.2 | 0.7-6.8 | 0.2 |
| Number of maintenance ASMs |  |  |  |
| 1  2  3  4 | 1.0  0.5  0.6  0.9 | 0.2-4.5  0.1-2.9  0.1-4.3  0.1-6.1 | 1.0  0.5  0.6  0.9 |
| Reasons for clinical encounter  New seizure  Increasing seizure/SE  Respiratory  Other | 2.1  2.6  6.3  1.5 | 0.3-14.3  0.5-12.3  0.7-55.3  0.3-8.6 | 0.5  0.2  0.1  0.6 |

^a^n = 81
